# Important Role of Bacterial Nucleoid-Associated Proteins in Discovery of Novel Secondary Metabolites

**DOI:** 10.3390/ijms26062393

**Published:** 2025-03-07

**Authors:** Xiulei Xia, Jihui Zhang, Jiazhen Zheng, Guojian Liao, Yanqin Ding, Yue Li

**Affiliations:** 1 State Key Laboratory of Microbial Diversity and Innovative Utilization, Institute of Microbiology, Chinese Academy of Sciences, Beijing 100101, China; 2 Shandong Engineering Research Center of Plant-Microbial Restoration for Saline-Alkali Land, College of Life Sciences, Shandong Agricultural University, Tai’an 271018, China; 3 College of Pharmaceutical Sciences, Southwest University, Chongqing 400715, China

**Keywords:** *Streptomyces*, antibiotics, nucleoid-associated proteins, secondary metabolites, biosynthesis, cryptic BGCs

## Abstract

Microbial secondary metabolites (SMs) serve as the main source of natural antibiotics. Bioinformatics analyses reveal that multiple secondary metabolites biosynthetic gene clusters (BGCs) exist in the genomes of fungi and bacteria but the vast majority remains silent due to the control of intricate regulatory networks. An in-depth comprehension of these regulatory processes is required for the activation of cryptic gene clusters. Among them, the regulations at the proteomic level originating from epigenetic modifications and their correlations with secondary metabolite biosynthesis have gained increasing interest recently, especially the modifications on bacterial nucleoid-associated proteins. This article highlights the recent advances and important roles of bacterial nucleoid-associated proteins (NAPs) in the biosynthesis of SMs. Developing new tools around NAPs would be significant for the discovery of novel bioactive compounds in microbial resources.

## 1. Introduction

Actinomycetes are a valuable resource of SMs with extensive bioactivities, such as anti-infection, anti-cancer, anti-malarial, and anti-parasitic agents, which have been widely applied in clinical, agricultural, and animal husbandry fields [1,2,3]. More and more SM BGCs have been found due to the rapid development of genome sequencing, but most of them remain silent under general laboratory fermentation conditions. Among them, the regulatory genes involved in SM biosynthesis play an important role and their coding proteins are generally classified as pathway-specific regulators, global regulators, or pleiotropic regulators [4]. Thus, various strategies based on these regulations have been developed for the activation and expression of ‘cryptic BGCs’ to mine bioactive molecules for tackling pharmaceutical dilemmas, which include the genetic manipulation of regulatory genes, ribosome engineering, CRISPR/Cas9 gene editing system-mediated activation, reporter-guided strategy, heterologous expression of silent BGCs, co-culture, optimization of fermentation conditions and so on [5]. Recently, the epigenetic modification of specific proteins, such as histones or bacterial NAPs, has shown great potential for activating cryptic SM BGCs [6].

Epigenetics is the study of heritable and stable modifications in gene expression that occur in the structure of chromosomes rather than DNA sequences [7], such as DNA methylation, histone modification, and non-coding RNAs. Post-translational modifications (PTMs) of histones and NAPs engage in many life processes of microbes including antibiotic biosynthesis, which is what we are interested in. Strategies relating to the PTMs of fungal histones have been proposed and applied into the regulation of gene transcription and activation of SM biosynthesis [8]. In bacteria, NAPs exert the equivalent role of histones in fungi, but NAP modifications and their effects or mechanisms on secondary metabolisms are not as clear as those of fungal histones. This paper provides an overview of the recent progresses in epigenetic modifications of bacterial NAPs, especially including the role of NAPs in the activation of silent BGCs.

## 2. The Roles of Epigenetic Modification in the Production of SMs in Microorganisms

Epigenetic modifications exhibit the capacity to activate the expression of silent genes and promote the biosynthesis of natural metabolites by enhancing or de-repressing regulatory genes directly or indirectly. For DNA methylation, hypermethylation in the gene promoter region causes gene repression, while hypomethylation activates gene expression. The addition of 5-azacytidine as a methyltransferase inhibitor induces the biosyntheses of sclerotiorin, atlantinones, and ochrephilone in *Penicillium citreonigrum* [9]. Post-translational modifications of histone, especially methylation or acetylation, are also promising in terms of activating the silent biosynthetic pathway and gene clusters [10]. The impact of histone modifications on SM biosynthesis in eukaryotic fungi has been well proved, but, correspondingly, less is known about the effects of NAPs on SM production in prokaryotic bacteria. Histones from fungi (eukaryotic) and NAPs from bacteria (prokaryotic) can be modified by a series of acylations, which are catalyzed by acyltransferases or happen spontaneously, so the acetylation levels may be altered via improving the acyltransferases activity or inhibiting the activity of deacylases.

### 2.1. Features of Bacterial NAPs

**Figure 1:**
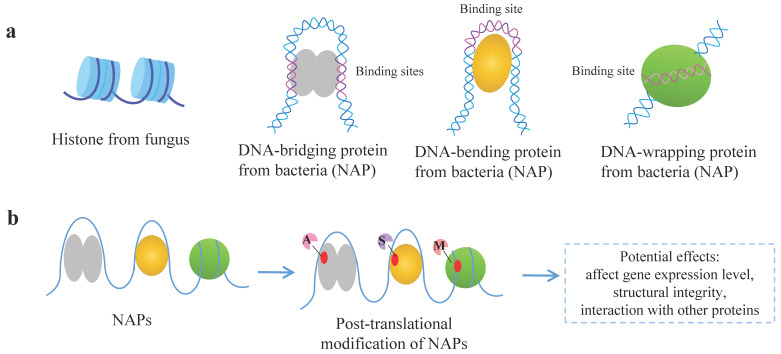
Features of NAP and its modes of action. (**a**) DNA-binding modes of NAPs. Histone from fungus as a comparison, DNA-bridging protein, DNA-bending protein, and DNA-wrapping protein as NAPs from bacteria are presented [12]. (**b**) Post-translational modification of NAPs. Figure was adapted from reference [16]. NAP, nucleoid-associated protein; A, acetylation; S, succinylation; M, methylation. Other modification types are described in Section 2.3.

Most of the genetic information of living creatures is stored in DNA, which is then transmitted and expressed through self-replication, transcription, and translation. To ensure the accuracy of genetic information, DNA needs to be stable and maintain a certain structure. In eukaryotic cells, including fungi, the binding of histone to DNA can facilitate the latter’s condensation into chromatin to affect DNA replication and chromosome segregation, and then regulate gene expression, while the nucleosomes serve as the basic unit of chromatin [7]. Whereas in bacteria, NAPs exert similar functions as the histones of eukaryotes, influencing the DNA structure, DNA repair, recombination, replication, and gene transcription [11,12]. They also contribute to the protection of DNA stability and functions for maintaining the integrity of genomic DNA through their capacity for non-specific DNA binding and DNA compaction [13,14]. Most NAPs binding to DNA can change the shape of bacterial DNA by bending, bridging, wrapping (Figure 1a), and polymerizing, thus making it more compact to affect gene transcriptions [12]. In addition, NAPs were reported to have the ability to regulate important phenotypic properties, including stress response and the virulence of pathogenic *Mycobacterium* and *Nocardia*, and regulate the secondary metabolism and differentiation in general actinomycetes [15]. Significantly, various types of PTMs of NAPs may perturb DNA-binding affinity, structural integrity, or protein–protein interactions [16] (Figure 1b).

The most important DNA-bridging protein representatives are the histone-like nucleoid structuring (H-NS) proteins from Gram-negative bacteria and another H-NS like NAP Lsr2 from Gram-positive bacteria, which can form a DNA–protein–DNA bridging structure. H-NS proteins as global regulators participate in the modulation of bacterial transcription by repressing 5–10% of genomic target genes [17]. The helix-turn-helix domain at the *c*-terminus preferentially binds to AT-rich DNA sequences, and the *n*-terminal domains presenting as dimers or higher-order dimer–dimer interactions as functional units would form complex oligomers binding to DNA, causing DNA compaction or gene silencing [18]. Lsr2 in *Mycobacterium tuberculosis* and other actinobacteria usually bind to AT-rich DNA to repress the transcription of numerous genes [19].

The functional properties of some bacterial NAPs have also been well studied, such as the integration host factor (IHF) with DNA bending ability, heat-unstable nucleoid protein (HU) with DNA bending and wrapping ability, and leucine-responsive regulatory protein (Lrp) with DNA wrapping and bridging ability [20,21]. The IHF functions similarly to traditional transcription factors, by attracting RNA polymerase to the promoter region. The resulting bending of the DNA can promote the contacting of regulatory proteins with RNA polymerase to affect transcription [12]. HU proteins preferentially bind to duplex DNA with a nick or a gap in a homodimeric form, causing DNA bending in a sequence-independent manner [18]. Factors for inversion stimulation (Fis) also belong to DNA-bending proteins and they present as homodimers consisting of 98 amino acids [22]. Another NAP, Lrp, has a broad influence on gene expression and usually presents with an octamer or large oligomers as the main functional unit, forming a disc-like structure to wrap DNA molecules and bring distant DNA regions closer, and thus resulting in DNA compaction [21]. Lrp can respond to the change of leucine concentration, causing the dissociation of Lrp–DNA complexes [23].

At present, the numbers of NAPs in bacteria are still increasing, including H-NS, Dps, StpA, CbpA, CbpB, EbfC, and MvaT in Gram-negative bacteria, and HupA, HupS, sIHF, mIHF, EspR, NapA, NapM, Dps-like proteins, and Gbn in Gram-positive bacteria [12,15,24,25]. Various types of NAPs have homologs in both G^+^ and G^‒^ bacteria, such as HupA (SCO2950) from *Streptomyces coelicolor* and HU protein from *E. coli* [25]. Some common NAPs in actinomycetes are summarized in Table 1.

**Figure 2:**
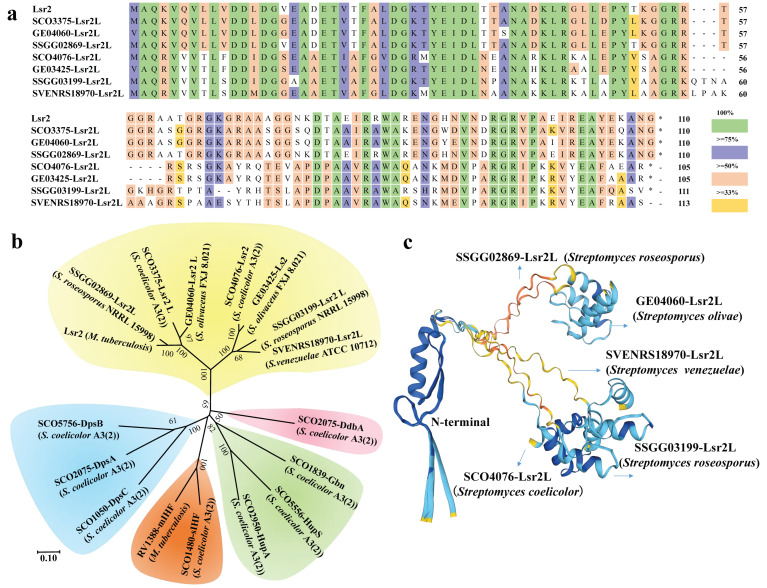
Amino acids sequence alignment of NAP Lsr2 in *Streptomyces*. (**a**). Sequence alignments of Lsr2 from *M. tuberculosis* with its homologues from *Streptomyces*: SCO3375, SCO4076 (from *S. coelicolor* A3(2)); GE04060, GE03425 (from *S. olivaceus* FXJ 8.021); SSGG02869, SSGG03199 (*S. roseosporus* NRRL 15998); and SVEN-RS18970 (from *S. venezuelae* ATCC 10712). Numbers indicate positions of amino acid residues from *n*-terminus of proteins. Identical amino acid residues are highlighted in green, and residue similarities with more than 75%, 50%, and 33% are, respectively, shown in purple, orange, and yellow. The asterisks represent the stop codon of proteins. (**b**). Phylogenetic tree of Lsr2 (*M. tuberculosis*) and its homologues from four *Streptomyces* strains. Tree was generated by software Mega 7 using Neighbor-Joining method. Evolutionary distances were computed using *p*-distance method and are in units of number of amino acid differences per site. Branches in tree are divided into five parts. (**c**). 3D structure alignment of Lsr2-type NAPs. Analysis was performed by SWISS-MODEL. The different colors represent the consistency among these protein sequences.

Since Lsr2 is an important and typical NAP in actinomycetes, we have collected seven sequences of Lsr2L amino acids from several *Streptomyces* and performed a sequence alignment with them. It was revealed that Lsr2 from *M. tuberculosis* has a high identity with SCO3375 and SCO4076 from *S. coelicolor* A3(2), GE04060 and GE03425 from *S. olivaceus* FXJ 8.021, SSGG02869 and SSGG03199 from *S. roseosporus* NRRL 15998, SVEN-RS18970 from *S. venezuelae* ATCC 10712 with a total identity of 67.83% (Figure 2a). The phylogenetic tree shows that Lsr2-type NAPs also have closer evolutionary relationships with other types of NAPs, exemplified by Dps-like proteins, IHF, Hup, Gbn, and DdbA (Figure 2b). In addition, 3D structures of five representative Lsr2L from four *Streptomyces* strains were established using SWISS-MODEL (https://swissmodel.expasy.org/). Their *n*-terminal domain is nearly the same, while significant differences in *c*-terminal conformation are indicated (Figure 2c).

### 2.2. Role of NAPs in Chromosome Dynamics During Streptomyces Life Cycles

*Streptomyces* has a unique and complex life cycle that includes spore germination, vegetative growth, aerial hyphal growth (early and late stages), spore maturation, release, and exploratory growth in some species (e.g., *S. venezuelae*). *Streptomyces* chromosomes, like those of fungus and other eukaryotic cells, rearrange themselves from ‘open’ to ’closed’ conformation. DNA-organizing proteins, topoisomerases, and NAPs are responsible for maintaining the conformation of nucleoids [35]. Many NAPs, including HupA, Lsr2, and sIHF, are generated during *Streptomyces* vegetative growth [15,36]. In *S. lividans*, a *hupA* deletion slows vegetative growth. During sporulation, NAPs, such as DpsA, HupS, and DdbA, are significantly expressed [33,34,37]. The *dpsA* mutant of *Streptomyces coelicolor* participates in chromosome segregation and exhibits variation in spore size and nucleoid volume [33]. Compared to Dps proteins, the deletion of the highly conserved *ddbA* in *S. coelicolor* impacts chromosome compaction as well as spore resistance to osmotic and oxidative stress [34]. Coincidentally, the *hupS* mutant of *S. coelicolor* displays increased nucleoid size and decreased chromosome compaction and spore resistance to heat stress [37]. Oppositely, *dpsC* and *dpsB* mutants have increased nucleoid compaction. Additionally, NAP sIHF can promote nucleoid condensation during sporulation [38,39]. In *S. venezuelae*, HupS coordinates with the DNA-organizing protein SMC to generate impacts on chromosome compaction and cell division [40]. These findings clarify the important roles of NAPs in the vegetative growth and sporulation of *Streptomyces*, as well as in the dynamics of chromosome compaction and segregation during the *Streptomyces* life cycle.

### 2.3. PTMs of Bacterial NAPs

The PTMs of bacterial NAPs include phosphorylation, methylation, glycosylation, sumolyation, biotinylation, ubiquitination, lipidation, and diverse acylations, such as acetylation, malonylation, succinylation, and crotonylation [41]. They have similar effects as the PTMs of fungal histones, and mainly modulate bacterial metabolism and physiological functions by affecting the chemical features of amino acid residues and their neighboring regions, hence altering the physicochemical features, structures, and stability of the proteins [9,42].

Mass spectrometry-based proteomic methods have demonstrated that extensive and diverse PTMs frequently occur in the most abundant and well-characterized NAPs, H-NS, IHF, HU, and FIS in *Escherichia coli* (Gram-negative bacteria), which can affect protein-protein interactions, oligomerization, and DNA-binding affinity [16]. Taking H-NS as an example, Lys83 and Lys87 of the H-NS linker region can be acetylated, which would obstruct the electrostatic interaction between lysine (positive charge) and DNA (negative charge) and reduce the DNA-binding properties. The succinylation of Lys6 imposes large carbon moieties onto the H-NS protein to induce sterical hindrance, and thus decrease H-NS’s binding affinity to DNA [16]. PTMs can also disturb NAP oligomerization. H-NS Tyr61 contributed to a hydrophobic core, which stabilizes the tail-to-tail connection of two H-NS dimers. Proteomic investigations revealed that the Tyr61 phosphorylation was negatively charged, rendering the region more hydrophilic and thus perturbing the oligomerization of the H-NS [43].

In comparison, PTMs of actinomycetes’ NAPs play considerable roles in DNA structuring and exert important regulatory functions [44]. Acetylation, phosphorylation, crotonylation, and succinylation often occur in the NAPs of actinomycetes or their related proteins. For instance, in *Streptomyces roseosporus*, Lys13 and Lys86 of the HU-β subunit can be acetylated, which plays a crucial role in the growth, development, and metabolic process of cells [45]. In *S. coelicolor*, the phosphorylation of Ser4 of SCO2950 (HupA-like) affects its primary metabolic processes [46]. Previous investigations reported that the lysine of some NAP-related proteins from *Streptomyces* can undergo crotonylation and succinylation [47,48]. Currently, although only a few studies about the PTMs of NAP in actinomyctetes have been reported, their importance and effects on the production or activation of SMs cannot be ignored.

## 3. The Acetylation of NAP as a Primary PTM Modification

Acylation modification, especially acetylation, is one of the primary PTMs of NAPs or histones. Fungal histone modification is a good reference to study the PTMs of NAP in bacteria. It was reported that histone acetylation has a major influence on the biosynthesis of some fungal metabolites [49]. It frequently occurs at positively charged lysine residues, which can weaken the interaction between DNA and histones, leading to a less compact chromatin structure to facilitate transcriptions [50]. The acetylation and deacetylation are usually kept in a dynamic equilibrium and controlled by histone acetyltransferases (HATs) and histone deacetylases (HDACs), respectively. The acetyl group of acetyl-coenzyme A is transferred by HATs to a specific lysine residue mainly situated at the *n*-terminus of histone. The negatively charged acetylation group on the histone can repel the negatively charged DNA, causing the DNA to become slacked to facilitate transcription factors initiating gene expression. On the contrary, HDACs can promote histones binding to the negatively charged DNA, resulting in more compact nucleosomes, which could prevent the binding of transcription factors to DNA, therefore affecting the expression of genes or BGCs [51]. HDAC inactivation or inhibition is thought to be an effective approach for activating the silent genes in SM pathways and the production of diverse bioactive compounds. In *Aspergillus nidulans*, the inactivation of HDAC caused the overexpression of SM genes, leading to an increased biosynthesis of sterigmatocystin, penicillin, or terrequinone A [52].

The functions of NAPs in bacteria are thought to be similar to those of histones, including their modification types, DNA-binding ability, and their effects on DNA compaction, sporulation, and antibiotics production. The NAP HBsu from *Bacillus subtilis* is acetylated at seven sites, regulating DNA compaction and developmental differentiation [53]. Lysine acetylase directly acetylates a HU family protein from *B. subtilis*, which was speculated to be broadly present in bacteria. It was reported that the NAP HupB from *M. tuberculosis* could be acetylated by Eis (enhanced intracellular survival) acetyltransferase or deacetylated by the NAD^+^ dependent deacetylase Rv1151c, as these enzymes are involved in the regulation of the acetylation level to cope with environmental changes [54]. Specifically, NAPs in Actinobacteria, e.g., Hup proteins (from *Mycobacterium tuberculosis*, *Saccharopolyspora erythraea*, and *Streptomyces roseosporus*), contain a unique *c*-terminal extension that is rich in basic amino acids. For example, HupB contains an additional *c*-terminal histone tail-like lysine-rich region, which can be an acetylation site and is modulated by some regulators or inhibitors [54]. In *S. venezuelae*, the acetylation level of lysine residues of the NAP HupS is controlled by deacetylase CobB1, which affects HupS binding to DNA and spore maturation [55]. Above all, the acetylation of NAPs or improving the acetylation level through overexpressing the acetyltransferases coding genes is essential for gene activation.

## 4. Effects of PTMs of Bacterial NAPs on Production of SMs

In bacteria, NAPs and their diverse PTMs, such as acetylation, phosphorylation, methylation, etc., can affect not only protein structures, but also influence enzyme functions (such as protein interactions, solubility, stability, folding and localization, receptor activation, cellular interactions, molecular transport, cellular metabolism, and signaling pathways). These modifications play important roles in the regulation of the morphological differentiation of actinomycetes (including cell growth and development, morphological differentiation, biofilm formation, cell signal transduction, dormancy, virulence, and antibiotic resistance). Actinomycetes are metabolically gifted bacteria, so we have a particular interest in the relationships of NAPs with antibiotics’ biosynthesis [56].

**Figure 3:**
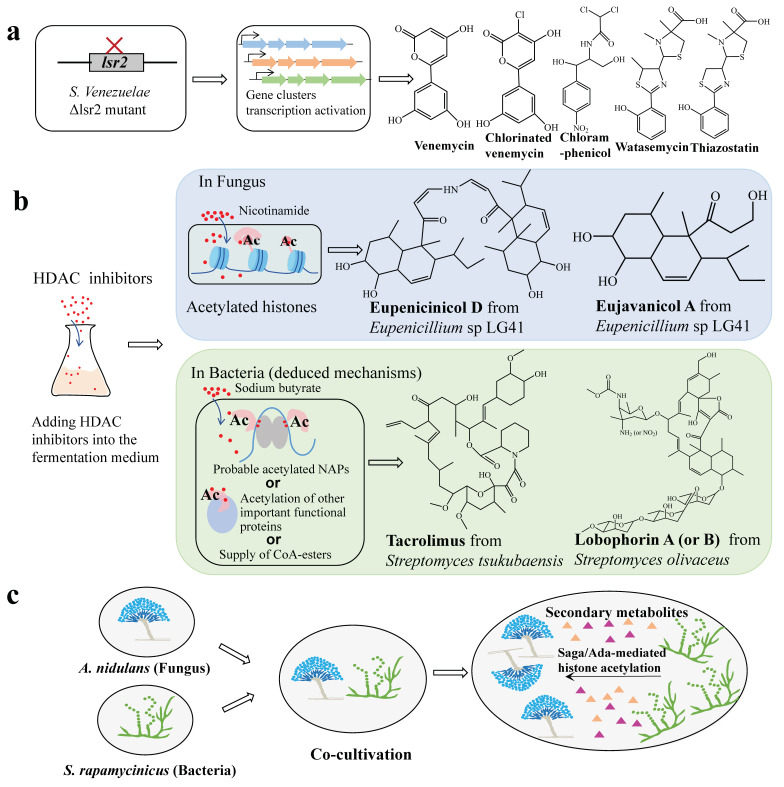
Post-translational modifications of proteins resulted in activation of SMs. (**a**). Disruption of NAP coding gene *lsr2* from *Streptomyces venezuelae* led to activation of SMs, such as venemycin, chlorinated venemycin, chloramphenicol, watasemycin, and thiazostatin. ‘X’ represents the deletion of lsr2 and the colorful arrows represent gene clusters. (**b**). Effects of HDAC inhibitors on SM production. Addition of HDAC inhibitors resulted in elevated levels of histone acetylation in fungus, or acetylation of probable NAPs or other important functional proteins in bacteria (deduced mechanisms). Meanwhile, it cannot be excluded that the accumulation of substantial butyrylphosphate or CoA-esters may be another important factor for the biosynthesis of natural products in *Streptomyces* treated by sodium butyrate. This process led to increased production of multiple SMs in *Eupenicillium* sp LG41 and two *Streptomyces* strains, respectively. (**c**). Co-cultivation of *Aspergillus nidulans* with *Streptomyces rapamycinicus* specifically activated the expression of cryptic fungal SM BGCs, leading to production of orsellinic acid and its derivatives. Co-cultivation involved histone H3K9 and H3K14 acetylation mediated by Saga/Ada complex, which contains histone acetyltransferase GcnE and AdaB protein required for activation of orsellinic acid BGC and other SMs. Triangles represent natural SMs. Adapted from Gehrke et al. [28], Li et al. [57], Wang et al. [58], Zheng et al. [59], and Nützmann et al. [60].

Various strategies targeting NAP coding genes or the PTM levels have been implemented to induce the activation of cryptic SMs. In *S. roseosporus*, Lsr2 also acts as a negative regulator inhibiting the biosynthesis of SMs, and the deletion of *lsr2* resulted in the production of pyrismycins A-F and six new sulfur-containing compounds, some of which have better bioactivities against certain human cancer cell lines [30]. In addition, twelve identified chemicals and a novel sesquiterpenoid, roseosporol A, were discovered from the Δ*lsr*2 strain of *S. roseosporus* [29]. Coincidentally, venemycin, CI-venemycin, thiazostatin, and watasemycin in the *lsr2* mutant of *S. venezuelae* were observed (Figure 3a). The researchers also found that several highly conserved clusters corresponding to the biosynthesis of siderophore/desferrioxamine and bacteriocin were directly controlled by Lsr2 [28], suggesting its pleiotropic regulatory role in *Streptomyces*. The down-regulation of Lsr2 may be an effective method to relieve metabolic repression, and radically affect the metabolic output of *Streptomyces*.

SCO1480 from *Streptomyces coelicolor* with high sequence homology to mIHF is another highly conserved NAP. It can bind to the promoter regions of the *actII-ORF4* and *redD* genes encoding the pathway-specific activators, thus affecting the biosynthesis of actinorhodin (ACT) and undecylprodigiosin (RED) [32]. Beyond that, the deletion of sIHF causes abnormalities in chromosomal compaction and segregation, and spore septum placement [38]. Also, a reduced production of blue-pigmented antibiotic actinorhodin was observed in *gbn* (*sco1839*) disruption mutant, representing a novel family of actinobacterial NAP [24].

Although some examples of the regulatory relationship between NAPs and antibiotic biosynthesis in actinomycetes have been reported, the underlying molecular mechanisms and the association of NAP PTMs with SM production are still poorly understood compared with those of fungal histones. More investigations should be carried out in this field to unveil the mystery of bacterial NAPs.

## 5. Activation of Silent BGCs by Adding Small Molecular Modifiers

Due to the importance of acyltransferase and deacylase in the regulation of protein PTMs, small molecule modifiers, as well as other epigenetic modifiers targeting these enzymes, are used to activate the silent SM BGCs. In fungi, numerous epigenetic modifiers exhibited essential roles in activating silent genes and increasing the production of natural metabolites [61]. They were preliminarily used to induce the production of various bioactive compounds, such as 5-azacytidine, suberoylanilide hydroxamic acid (SAHA), RG-108, suberohydroxamic acid (SBHA), nicotinamide, valproic acid (VA), sodium butyrate (SB), trichostatin A (TSA), and others [62]. By using methyltransferase inhibitor 5-azacytidine, *Cochliobolus lunatus* produced seven new diethylene glycol phthalate ester monomers and oligomers, while *Pestalotiopsis crassiuscula* produced a new coumarin [63,64]. In comparison, HDAC inhibitors were used more widely, which can be classified into four types including hydroxamic acids, cyclic peptides, short-chain fatty acids, and benzamides [65]. The treatment of the NAD^+^-dependent HDAC inhibitor, nicotinamide, increased the yield of two new decalin-containing compounds, eupenicinicols C and D, in fungus *Eupenicillium*, as well as other related compounds, such as eujavanicol A and eupenicinicols A (Figure 3b) [57]. A review has summarized the detailed progresses in this field [66], including the effects of sodium butyrate on activating or enhancing the production of approximately 40 natural compounds in fungi, with concentrations ranging from 9 µM to 10 mM.

Up to date, the impact of HDAC inhibitors on SM biosynthesis in prokaryotes, especially in *Streptomyces*, remains an interesting topic. Our published study revealed that the addition of sodium butyrate as a HDAC inhibitor can activate the expression of lobophorin BGCs in *Streptomyces olivaceus* FXJ 8.021 (Figure 3b). The acetylomics revealed that acetylation levels were increased at 218 sites in 190 proteins and decreased at 411 sites in 310 proteins, including those involved in transcription/translation or in lobophorin biosynthesis [59]. Additionally, three chemical elicitors, sodium butyrate (SB), dimethylsulfoxide (DMSO), and LaCl_3_ could increase tacrolimus accumulation in *Streptomyces tsukubaensis* (Figure 3b) [58]. Metabolomics analysis revealed that the central carbon metabolism, amino acid metabolism, and fatty acid metabolism showed significant differences, and also the supply of CoA was strengthened. Presumably, these changes could contribute to the yield improvement of tacrolimus. Overall, we deduced that sodium butyrate may be a useful elicitor of cryptic BGCs in bacteria; although, direct evidence that they affect SM biosynthesis via the PTMs of NAPs is lacking and the mechanisms remain unclear presently. By referring to histone function in fungus, we assumed that NAPs in bacteria might be similarly less specific to DNA with low affinity, and its effects could be pleiotropic. Alternatively, these inhibitors might exert their effects via other mechanisms, including the acetylation of certain key functional proteins involved in SM production, such as some unknown regulators, translational machinery components, key enzymes, and also probably the enhanced biosynthesis of CoA-ester or butyrylphosphate. In a situation where a high concentration of inhibitors is needed, the mechanism could be more complicated, especially considering the real-time intracellular concentration of these substances, which can be variable depending on the uptake efficiency of individual cells under specific circumstances. A more plausible suggestion is to screen different types of chemical modifiers prior to carrying out in-depth investigations. Among them, many potent HDAC inhibitors are available, such as vorinostat, panobinostat, tucidinostat, romidepsin, belinostat, etc.

In addition, the co-culture of different microorganisms is another efficient strategy to activate SMs, since some chemical molecules might be released from one microorganism and function as modifiers to trigger the production of active compounds in the other microorganism. It was found that *Streptomyces rapamycinicus* induced *Aspergillus nidulans* to produce orsellinic acid and its derivatives, which was ascribed to the histone H3K9 and H3K14 acetylation of *A. nidulans* via the Saga/Ada complex containing histone acetyltransferase GcnE and AdaB protein. Also, the Saga/Ada complex plays a major role in the regulation of SM productions (Figure 3c) [60]. Another similar histone modification example appeared in the co-cultivation of *A. fumigatus* and *S. rapamycinicus*, resulting in the production of fungal SMs fumicyclines A and B [67]. Elucidating the interacting mechanisms between different microorganisms may provide a new avenue for acquiring cryptic metabolites from actinomycetes.

Overall, acetyltransferase and deacetylase inhibitors provided pipelines to mine new antimicrobial agents or affect the developmental differentiation via modifying acetylation levels. Using small-molecule epigenetic modifiers to discover novel bioactive compounds of *Streptomyces* and other bacteria is promising, while discovering more effective small-molecule epigenetic modifiers would be significant.

## 6. Conclusions

In summary, PTMs of both eukaryotic histones and bacterial NAPs are experiencing rapid development and have become effective targets of engineering to trigger the activation of cryptic SM BGCs or improve the yield of antibiotics. However, since the regulation of NAPs on SM biosynthesis usually involves multiple pathways and various regulator-mediated hierarchical cascades, rather than a point-to-point direct control, there are some key issues to be considered. (1) In terms of the modification types of NAPs, modulating the acylation level is one of the common approaches as the related enzymes have been defined, and their inhibitors and activators are easily accessible, despite some limitations being caused by their poor stability and permeability into cells might exist. The genetic manipulation of the coding genes is also acceptable by using various methods, such as CRISPR editing, CRISPR interference (CRISPRi), and CRISPR activation (CRISPRa). With the advance in AI technology, computing-based simulation would facilitate the re-design of a NAP structure to modulate its binding to DNA or regulatory function. (2) For determining the SM molecules, traditional LC-HR-MS analysis is a general approach. Alternatively, if the regulatory pathway of a certain NAP on the target SM is clear, then modulating the PTM level of this protein could be more straightforward. In addition, the rational establishment of the global correlations of NAPs with SMs by using genomics, proteomics, and metabolomics, as well as other emerging products-mining tools, would be promising. (3) Finally, the appropriate combination of the above approaches with other technologies, including artificial intelligence, machine learning, and deep learning technologies, and co-culturing different microorganisms, would provide new pipelines for drug discovery in the future. Overall, since bacteria serve as important resources of new drugs, further in-depth study of the epigenetics of NAPs would not only enrich and promote our understanding on protein modifications, but also greatly contribute to the activation of silent SM BGCs to discover more specialized metabolites as potential drug leads.

## Figures and Tables

**Table 1:** Some common types of NAPs in actinomycetes.

NAP Types	Proteins	Strain Resources (Example)	Effects on SMs Production	References
Lsr2	SVEN-RS18970, SSGG02869, SSGG03199, GE04060, GE03425, SCO3375, SCO4076,	*Streptomyces venezuelae*, *Streptomyces* sp. *WAC07094*, *Streptomyces roseosporus*, *Streptomyces olivaceus*, *Streptomyces coelicolor*	Chloramphenicol/Venemycin/CI-venemycin/Thiazostatin/Watasemycin, Saquayamycin, Roseosporol A/pyrismycins A-F, Lobophorins	[26,27,28,29,30]
HupA/ HupS	SCO2950, SCO5556	*Streptomyces coelicolor*	Carotenoid compounds, undecyloprodigiosin, ectoine,	[31]
sIHF	SCO1480	*Streptomyces coelicolor*	Actinorhodin, undecylprodigiosin	[32]
Gbn	SCO1839	*Streptomyces coelicolor*	Actinorhodin, sporulation	[24]
Dps-like proteins	SCO0596, SCO5756, SCO1050	*Streptomyces coelicolor*	Condensation of spore nucleoids	[33]
DdbA	SCO2075	*Streptomyces coelicolor*	Modulate RNA polymerase activity combined with ppGpp, organize DNA conformation in response to stress	[34]

## Abbreviations

SMs: secondary metabolites; BGCs: biosynthetic gene clusters; NAPs: nucleoid-associated proteins; PTMs: protein post-translational modifications; HAT: histone acetyltransferase; HDAC: histone deacetylase; Lys: lysine; H-NS: histone-like nucleoid structuring protein; IHF: integration host factor; HU: heat-unstable nucleoid protein; Lrp: leucine-responsive regulatory protein; SAHA: suberoylanilide hydroxamic acid; CbpA, curved-DNA-binding protein A; CbpB, curved-DNA-binding protein B (also known as Rob); Dps, DNA protection from starvation; Fis, factor for inversion stimulation.
